# Alignment Prediction of Air Spring Vibration Isolation System for Marine Shafting Based on Genetic Algorithm–Back Propagation Neural Network

**DOI:** 10.3390/s24134049

**Published:** 2024-06-21

**Authors:** Song Liu, Liang Shi, Zhi-Wei Liu

**Affiliations:** 1 Institute of Noise and Vibration, Naval University of Engineering, Wuhan 430033, China; 2 National Key Laboratory on Ship Vibration & Noise, Wuhan 430033, China

**Keywords:** alignment prediction, back propagation (BP), neural network (NN), genetic algorithm (GA), air spring vibration isolation system (ASVIS), marine shafting

## Abstract

Shafting alignment plays an important role in the marine propulsion system, which affects the safety and stability of ship operation. Air spring vibration isolation systems (ASVISs) for marine shafting can not only reduce mechanical noise but also help control alignment state by actively adjusting air spring pressures. Alignment prediction is the first and a key step in the alignment control of ASVISs. However, in large-scale ASVISs, due to factors such as strong interference and raft deformation, alignment prediction faces problems such as alignment measurement sensors failure and difficulty in establishing a mathematical model. To address this problem, a data model for predicting alignment state is developed based on a back propagation (BP) neural network, fully taking advantage of its self-learning and self-adaption abilities. The proposed model exploits the collected data in the ASVIS instead of the alignment measurement data to calculate the alignment state, providing another alignment prediction approach. Then, in order to solve the local optimum issue of BP neural network, we introduce the genetic algorithm (GA) to optimize the weights and thresholds of the BP neural network, and an improved GA-BP model is designed. The GA-BP model can leverage the advantages of the global search capability of GA as well as the BP neural network’s fast convergence in local search. Finally, we conduct experiments on a real ASVIS and evaluate the prediction models using different criteria. The experimental results show that the proposed prediction model with the GA-BP neural network can accurately predict the alignment state, with a mean-square error (MSE) of 0.0114. And compared to the BP neural network, the GA-BP neural network reduces the MSE by approximately 74%.

## 1. Introduction

The air spring vibration isolation system (ASVIS) can significantly reduce mechanical noise while providing high-precision attitude control capabilities, and has been widely used in vessels and high-speed railways [1,2,3]. But for the air spring vibration isolation system of marine propulsion devices, external disturbances such as ship tilting and changes in operational conditions may result in shaft misalignment, which may affect the operational safety of the propulsion device and attenuate the vibration isolation effect [3,4]. Thus, it is necessary to monitor and predict the alignment state in real time, and carry out precise alignment control by actively adjusting the air spring pressure. However, in large-scale ASVISs, due to factors such as strong interference and raft deformation, alignment prediction faces problems such as alignment measurement sensors failure and difficulty in establishing a mathematical model [5,6], making alignment prediction a challenging problem.

The first issue is how to measure the alignment state. It can be roughly divided into the direct measurement method and indirect measurement method [7,8,9,10]. Currently, indirect measurement methods are widely used in the ASVIS [11]. In this method, the height difference at different positions of the ASVIS is first measured, and then the alignment state is calculated using a given mathematical formula. And eddy current sensors are typically employed to measure the height differences due to their stable operation and high reliability in engineering [12]. However, these sensors are prone to electromagnetic interference, which can cause disturbances or deviations in the measured values, consequently impacting the accuracy of alignment predictions. Compared to eddy current sensors, pressure sensors convert mechanical pressure in gases or liquids into electrical signals, offering enhanced resistance to interference [13]. In fact, research has found that there is an approximately linear mapping relationship between the internal pressure of an air spring isolator and its bearing capacity when the height of the air spring isolator remains constant [14,15]. Thus, intuitively speaking, there may also be a certain correlation between the air spring pressure and alignment state. But to date, we have not yet discovered any studies on predicting the alignment status using air spring pressure values.

The second issue is how to calculate the alignment state. Existing models for calculating alignment states typically assume an ideal rigid body model for the raft, and a static mathematical model can be obtained based on parameters such as sensor deployment position [3,9]. However, large air spring isolation systems, like whole cabin vibration isolation systems, exhibit nonlinear and time-varying characteristics [11]. On one hand, these systems carry multiple loads and operate under complex combined conditions, including propulsion equipment, producing various torques at different speeds, causing dynamic changes in the load-bearing capacity of air spring isolators at different positions. On the other hand, the large rafts of these isolation systems span large distances, leading to raft deformations that may also change with different operational conditions. In this case, it is difficult to accurately predict the alignment state using traditional mathematical models, and it is necessary to explore novel alignment prediction models to address this issue.

With the advancement in artificial intelligent technology, back propagation (BP) neural networks have introduced a new approach for complex system modeling [16,17,18]. The BP neural network (NN) is composed of a large number of neurons. By imitating the biological nervous system, the BP neural network extracts useful knowledge from the collected data and completes the model construction of the nonlinear system adaptively. And it has been widely used for fault detection, function approximation, structure optimization, and so on. Nevertheless, the BP neural network also has its own shortcomings, that is, it is apt to fall into the local optimum. To solve this problem, scholars have put forward many suggestions. Among them, swarm intelligent optimization algorithms, such as the genetic algorithm (GA) and particle swarm optimization (PSO) [19,20], are used to optimize the parameters of the BP neural network, which effectively improves the accuracy of the algorithm.

In this paper, we propose an alignment prediction model for the air spring vibration isolation system based on the BP neural network. By fully exploiting the self-learning and self-adapting capabilities of the BP neural network, the data mapping relationship between air spring pressures and the alignment state in an ASVIS is constructed. In order to overcome the local optimum issue and obtain the global optimum, the genetic algorithm is introduced for optimizing the parameters of the BP neural network, and an improved GA-BP model is designed. Ideally, the GA-BP model can make full use of the global search ability of the genetic algorithm and the fast convergence of the BP neural network in local search. To verify the effectiveness of the proposed models, we conduct experiments on a real ASVIS and evaluate the prediction model using four typical criteria. Extensive experimental results reveal that our proposed prediction model based on the GA-BP neural network can accurately and effectively predict the alignment state, with an average accuracy of 98.7%, a determinant coefficient ($R^{2}$) of 0.9539, a mean absolute error (MAE) of 0.0725, and a mean-square error (MSE) of 0.0114. And compared to the BP model, the GA-BP model improves the accuracy and $R^{2}$ by approximately 1.1% and 12%, respectively, and reduces the MAE and MSE by approximately 38% and 74%, respectively. In the future, the alignment prediction model can be used independently or deployed incrementally in existing systems. For example, existing ASVISs usually use eddy current sensors to monitor their attitude. However, when the sensor fails and the alignment state cannot be calculated directly, we can still use the air spring pressures to predict the alignment state so as to improve the reliability of the system.

The main contributions of this paper can be summarized as follows:

- To the best of our knowledge, we are the first to study the data mapping relationship between air spring pressure and alignment state in an air spring vibration isolation system. And we propose a data model based on the BP neural network to predict the alignment state.
- We applied the genetic algorithm to optimize the weights and thresholds of the BP neural network and introduced an improved GA-BP prediction model. The model makes full use of the local search ability of the BP neural network as well as the global search ability of the genetic algorithm, through which the prediction accuracy is increased.
- Extensive experiments are conducted to validate the effectiveness and efficiency of our proposed models. The results show that the prediction model, based on the BP and GA-BP neural networks, can accurately predict the alignment state. And compared with the BP model, the GA-BP model can obtain higher alignment prediction accuracy.

The rest of this paper is organized as follows. Section 2 briefly describes the system model of ASVIS. Section 3 introduces the alignment prediction model based on the BP neural network and the improved GA-BP algorithm. In Section 4, we conduct experiment with a real ASVIS and present our evaluation results. Finally, the paper is concluded in Section 5.

## 2. System Model

**Figure 1:**
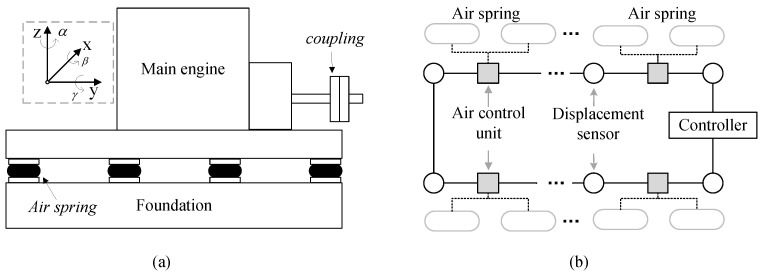
The composition and connection diagram of ASVIS. (**a**) the schematic diagram of ASVIS and coordinate system. (**b**) the electrical connection diagram of ASVIS.

An air spring vibration isolation system mainly consists of air springs, an air control unit (ACU), displacement sensors, and a controller as shown in Figure 1. Air springs are used to isolate mechanical vibrations and support equipment, such as the main engine, as shown in Figure 1a. The ACU can adjust the air spring pressure by inflating and deflating an air spring. The change in the pressure value of an air spring may change the height of the air spring, thus affecting the alignment state of the system. Displacement sensors are used to monitor the height of the ASVIS at different positions. The controller can calculate the alignment state using the monitored data of displacement sensors, and then generate optimized control strategies. The controller, ACUs, and displacement sensors are usually connected and interact with each other through an industrial bus as shown in Figure 1b. In addition, the controller can communicate with other devices to obtain operating condition data, such as the rotational speed of the main engine.

When the ship tilts or the output torque of the main engine changes, the force on the air spring changes accordingly, leading to a change in the air spring deformation and shaft alignment state. For the convenience of description, a coordinate system is established as shown in Figure 1a, and the output shaft of the main engine can be considered the positive direction of the *y*-axis. In engineering, the alignment state is usually described by four parameters, that is, horizontal angularity, vertical angularity, horizontal offset, and vertical offset. We take the coordinate values in the ideal alignment state as the origin. The alignment state vector in the coupling center point can be described as:

(1) $$\Gamma =\{x_{c},z_{c},\alpha ,\beta \}$$

in which $x_{c}$ is the horizontal offset in the *x*-axis, $z_{c}$ is the vertical offset in the *z*-axis, α is the horizontal angularity, and β is the vertical angularity.

## 3. The Design of Alignment Prediction Model

In this section, we first present the details of the alignment prediction model based on the BP NN, including the network structure, activation function, etc. Then, we introduce how to use the genetic algorithm to optimize the parameters of the NN, and the work flow of the improved prediction model based on the GA-BP NN.

### 3.1. The Prediction Model Based on BP NN

**Figure 2:**
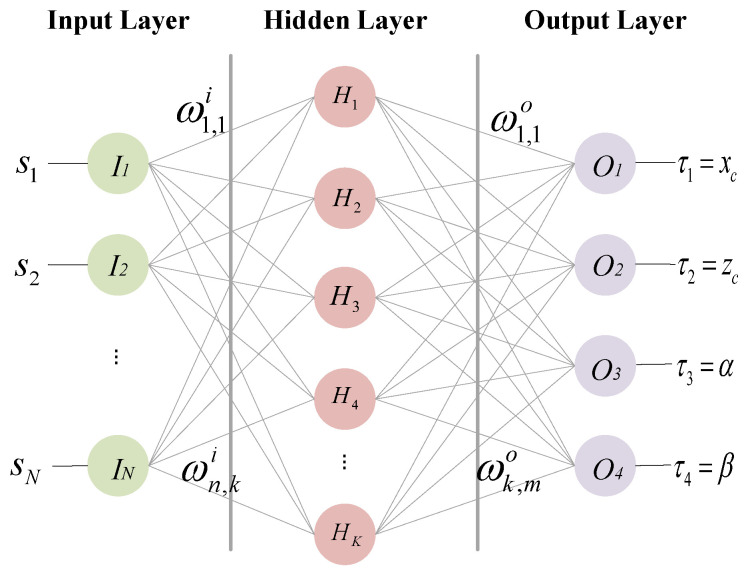
Alignment prediction model based on the BP NN.

A BP NN generally consists of an input layer, several hidden layers, and an output layer. This paper adopts a typical single hidden layer BP NN, and the topology structure is shown in Figure 2. $S=\{s_{1},...,s_{N}\}$ is the input vector; $I_{n}$ is the input layer; $H_{k}$ is the hidden layer; $O_{m}$ is the output layer; and $\tau_{m}$ is the output value of the output layer.

For values in the input vector *S*, $s_{i}$ (1≤i≤N) represents a system state value of the ASVIS, which can be the air spring pressure, the rotational speed of a main engine, and so on. In this paper, we focus on the data mapping relationship between the air spring pressure and alignment state. Thus, $s_{i}$ means the pressure value of the *i*-th air spring here. The output values represent the alignment state Γ as mentioned above.

Assuming that the nodes number in the input layer is *N* and the nodes number in the output layer is *M*, we can estimate the nodes number in the hidden layer by the following empirical formula:

(2) $$K=\sqrt{N+M}+a$$

where *a* is a constant, typically taking a value range of 0 to 10. For example, we have 8 air spring pressure values as inputs (*N* = 8) and 4 alignment state values as outputs (*M* = 4). Then, we can set the number of nodes *K* in the hidden layer between 4 and 13.

In a BP NN, each layer has plentiful neurons to mimic the biological nervous system. And a neuron can be seen as a linear function, which is composed of input values, weighted values, threshold values, and an output value. As shown in Figure 2, the weights and thresholds from the input layer to the hidden layer are represented as $\omega_{n,k}^{i}$ and $b_{k}^{i}$, respectively. The output value of the *k*-th neuron (1≤k≤K) in the hidden layer $net_{k}^{i}$ can be calculated by:

(3) $$net_{k}^{i}=\sum_{n=1}^{N}\omega_{n,k}^{i}s_{n}-b_{k}^{i}$$

where *n* and *k* are the neuron number in the input layer and hidden layer, respectively. Generally, the activation function is adopted in the hidden layer to enhance its ability to learn the complex nonlinear relationship. Here, we use the ReLU function as the activation function, which is defined as:

(4) f(x)=max(0,x)

Then, the output value of the *k*-th neuron in the hidden layer through the activation function $net_{k}^{h}$ can be written as:

(5) $$net_{k}^{h}=f\left(\sum_{n=1}^{N}\omega_{n,k}^{i}s_{n}-b_{k}^{i}\right)$$

The output value of the *m*-th (1≤m≤M) neuron in the output layer $net_{m}^{o}$ can be calculated by:

(6) $$net_{m}^{o}=\sum_{k=1}^{K}\omega_{k,m}^{o}net_{k}^{h}-b_{m}^{o}$$

where $\omega_{k,m}^{o}$ and $b_{m}^{o}$ are the weights and thresholds from the hidden layer to the output layer, respectively. According to Equations (5) and (6), the mapping relationship between the input value in *S* and the output value in Γ can be described as:

(7) $$\tau_{m}=net_{m}^{o}=\sum_{k=1}^{K}\omega_{k,m}^{o}net_{k}^{i}-b_{m}^{o}=\sum_{k=1}^{K}\left(\omega_{k,m}^{o}f\left(\sum_{n=1}^{N}\omega_{n,k}^{i}s_{n}-b_{k}^{i}\right)-b_{m}^{o}\right)$$

In the training process of the BP NN, it will compare the predicted value with the measured value to calculate an error using the input data and output data. If the error does not meet the requirements, it will back propagate the error from the output layer to the input layer. In this process, the weights and thresholds will be updated by the gradient descent method. Here, the error between predicted value and the measured value is defined as:

(8) $$E=\frac{1}{m}\sum_{i=1}^{m}{\left({y_{i}}^{\prime }-y_{i}\right)}^{2}$$

where ${y_{i}}^{\prime }$ is the predicted value, and $y_{i}$ is the measured value. Accordingly, the weights and thresholds can be updated by:

(9) $$w_{n,k}^{i}=w_{n,k}^{i}-\alpha \frac{dE}{dw_{n,k}^{i}}$$

(10) $$w_{k,m}^{o}=w_{k,m}^{o}-\alpha \frac{dE}{dw_{k,m}^{o}}$$

(11) $$b_{k}^{i}=b_{k}^{i}-\alpha \frac{dE}{db_{k}^{i}}$$

(12) $$b_{m}^{o}=b_{m}^{o}-\alpha \frac{dE}{db_{m}^{o}}$$

where α is the learning rate.

### 3.2. GA-BP NN Model

BP NNs can effectively characterize nonlinear mapping relationships and update the weights and thresholds based on the collected data by self-learning. However, the performance of the original BP NN is largely affected by the parameters’ initialization value and apt to fall into the local optimum. The genetic algorithm, which is based on the idea of biological evolution in nature, has good global searching ability and is suitable for dealing with complex nonlinear optimization problems. Thus, we introduce the genetic algorithm to optimize the weights and thresholds of the BP NN.

**Figure 3:**
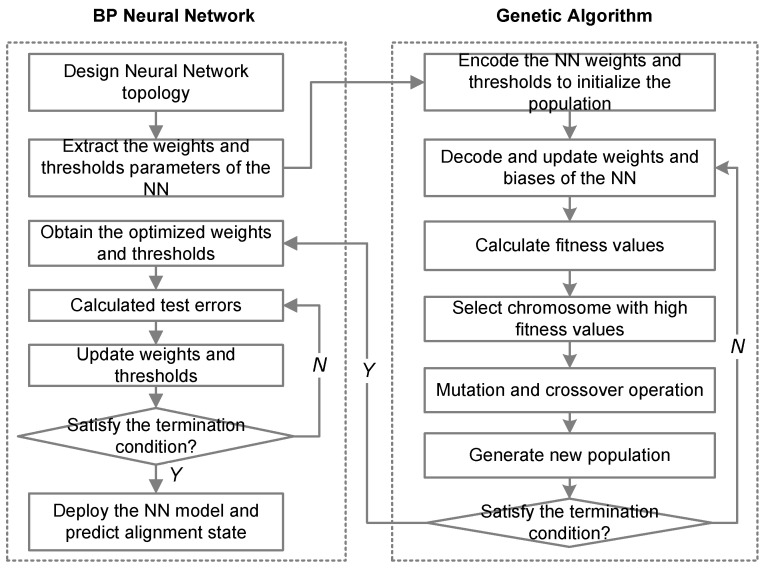
The work flow of the GA-BP NN model.

The GA-BP model can be roughly divided into two parts, the BP neural network and GA algorithm. The BP algorithm can quickly converge to the local optimal value, and the GA algorithm is capable of global optimization, but the convergence speed is slow when there are too many network parameters. Therefore, we hope to combine the advantages of both. First, we use the GA algorithm for global optimization. After reaching the preset goal, we update the parameters to the BP neural network. Then, the BP algorithm is used for further optimization. The work flow of the proposed GA-BP NN model is shown in Figure 3. The design of the BP NN for alignment prediction is described in Section 3.1. Here, we focus on the details of the GA algorithm.

(1) Encoding and population initialization: we use real numbers to represent the weights and thresholds of the BP NN, and encode these numbers into an array. The array can also be regarded as a chromosome, and elements in the array can be regarded as genes. Several chromosomes form a population. And the length of the chromosome is the total number of weights and thresholds in the BP NN, which can be calculated by:

(13) L=N×K+K+K×M+M

(2) Calculation of fitness function: The fitness function is used to evaluate the quality of chromosomes. Generally speaking, the larger the fitness value, the closer it is to the optimal solution. Since our goal is to minimize the error between the predicted value and the actual value, the fitness function here is expressed by the reciprocal of the MSE value:

(14) $$F=\frac{1}{E}=\frac{m}{\sum_{i=1}^{m}{\left(y_{i}-y_{i}^{\prime }\right)}^{2}}$$

(3) Selection. The higher the fitness value, the better the chromosome. Thus, we tend to select chromosomes with high fitness values. Here, we use the fitness proportionate selection method. The greater the fitness of the chromosomes, the greater the probability of being selected, and the probability $p_{i}$ of the *i*-th chromosome being selected can be calculated by:

(15) $$p_{i}=\frac{F_{i}}{\sum_{j=1}^{N_{pop}}F_{j}}$$

where $F_{i}$ is the fitness value of the *i*-th chromosome in the population. $N_{pop}$ is the number of the population.

(4) Crossover and mutation. Crossover refers to the exchange of genes at the same position between different chromosomes to form new chromosomes. This paper uses the uniform crossover method. Assume that the crossover probability is $p_{c}$. Then, for two different chromosomes, each gene of them has a probability of $p_{c}$ to exchange with each other, that is:

(16) $$\left\{\begin{array}{cc} c_{ik}=c_{jk}, & p>p_{c}\\ c_{jk}=c_{ik}, & p>p_{c} \end{array}\right.$$

where $c_{ik}$ and $c_{jk}$ are the *k*-th gene for the *i*-th chromosome and the *j*-th chromosome, respectively. *p* is a random value in [0,1].

Mutation means that some gene values randomly change to enrich the gene diversity. Suppose that the maximum value of mutation is $v_{r}$ and the probability of variation is $p_{m}$. When a gene is selected for mutation according to the probability $p_{m}$, the value after mutation is updated by:

(17) $$c_{ik}=c_{ik}+\phi \cdot v_{r}$$

where ϕ is a random value in [−1,1].

When the model training is complete, we can deploy the model based on the optimized parameters and perform alignment predictions.

## 4. Performance Evaluation

In this section, we evaluate the performance of the proposed prediction model based on the BP and GA-BP NNs and present the experimental results. Firstly, we experimented with a real air spring vibration isolation system and collected the operation data required for the model learning. Then, in order to verify the effectiveness of the GA-BP model, we compared the performance of GA-BP with original BP NN, in which four different criteria were adopted. Finally, we studied the impact of different parameters on the performance of our proposed model.

### 4.1. Experiment Setup

**Figure 4:**
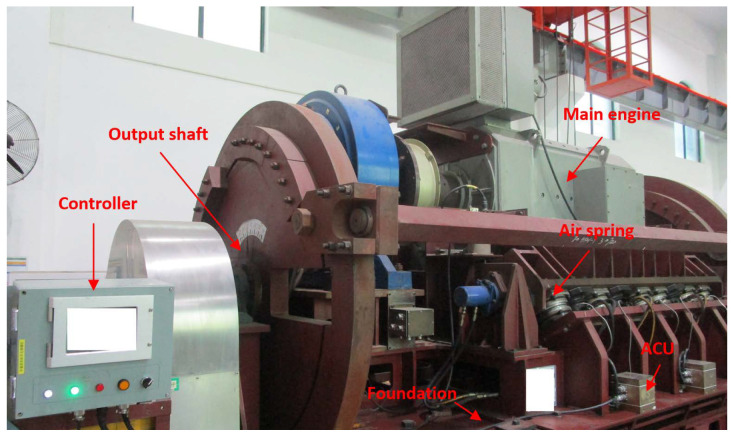
Physical diagram of ME-ASVIS used in the experiment.

**Figure 5:**
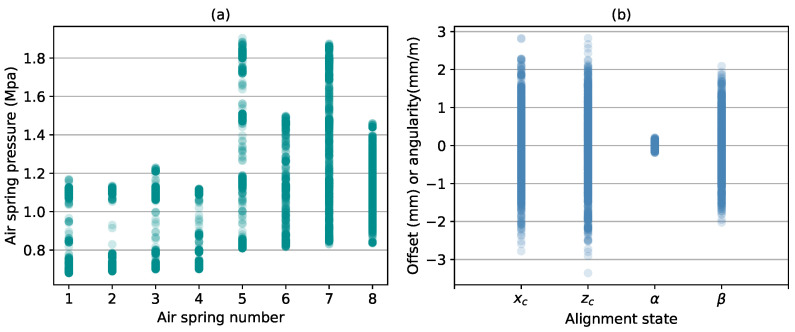
The value distribution of the collected data. (**a**) the distribution of air spring pressure values. (**b**) the distribution of the alignment state.

(1) Experimental device: In this paper, we carried out the experiment with a main engine air spring vibration isolation system (ME-ASVIS) as shown in Figure 4. In the ME-ASVIS, we use eight air springs to support the main engine with an inclined angle of 30°. Each air spring has a rated load of 8 tons and a natural frequency of 5 Hz. Air springs are connected to the ACUs to monitor its pressure and perform charging and deflating operations. The alignment state is monitored and calculated by seven displacement sensors. And these data are recorded and stored in the controller.

(2) Data collection: Within the acceptable working pressure range of the air spring and near the ideal alignment state, the air pressure is changed randomly through inflation and deflation. During this process, we monitor the air pressure values and alignment state. A total of 19,336 groups of data are recorded, where each group of data includes eight air spring pressure values and four alignment state values. The distribution of recorded data is shown in Figure 5. And we can see that the change in air spring pressure has little effect on horizontal angularity α. In addition, we randomly divide the data into a training set and test set, of which the training set accounts for 80% and the test set accounts for 20%.

(3) Model implementation: The proposed model is implemented by Python (version 3.12.2), and pytorch (version 2.2.1) is used to provide the basic function for establishing a neural network. The GPU is NVIDIA GeForce RTX 3070 (Santa Clara, CA, USA) and the CUDA version is 12.4.89. For BP NN, the learning rate is set to 0.02, the default number of nodes in the hidden layer is set to 8, and the Adam optimizer is selected in the BP algorithm. For GA, the default population is 50, the crossover probability is 0.5, the mutation probability is 0.1, and the maximum mutation value is 0.05. The maximum training iteration is 5000.

(4) Performance metrics: In order to demonstrate the effectiveness of the proposed model, in addition to MSE, three other typical criteria are used in the paper, that is, mean absolute error, determinant coefficient, and accuracy. The mean absolute value is expressed by:

(18) $$MAE=\frac{1}{n}\sum_{i=1}^{n}\left|{y_{i}}^{\prime }-y_{i}\right|$$

where *n* is the amount of collect data, ${y_{i}}^{\prime }$ is the *i*-th predicted value, and $y_{i}$ is the *i*-th measured value. And the determinant coefficient $R^{2}$ is defined as:

(19) $$R^{2}=1-\frac{SSE}{SST}=\frac{SSR}{SST}$$

in which SST is the total sum of squares, SSR is the regression sum of squares, and SSE is the residual sum of squares. These values can be calculated by:

(20) $$SST=\sum_{i=1}^{n}{\left(y_{i}-\bar{y}\;\right)}^{2}$$

(21) $$SSR=\sum_{i=1}^{n}{\left({y_{i}}^{\prime }-\bar{y}\;\right)}^{2}$$

(22) $$SSE=\sum_{i=1}^{n}{\left(y_{i}-{y_{i}}^{\prime }\right)}^{2}$$

where $\bar{y}$ is the average value of $y_{i}$.

In engineering, when the alignment state is within the given error range, we believe that the system is in the alignment state and can stop the alignment control; otherwise, it is considered that the system is in the misalignment state and alignment control needs to be continued. Here, we set the alignment error interval to 0.3, that is, when $|x_{c}|\;<0.3$ mm, $|z_{c}|\;<0.3$ mm, |α|<0.3 mm/m, and |β|<0.3 mm/m, the system is considered to be in the alignment state. Then, the accuracy of the alignment prediction model is defined as:

(23) $$Accuracy=\frac{TP+TN}{N_{total}}$$

where $N_{total}$ is the total number of samples in the test set, TP is the sample amount such that the predicted alignment state is the actual alignment state, and TN is the sample amount such that the predicted misalignment state is the actual misalignment state.

### 4.2. Results

**Figure 6:**
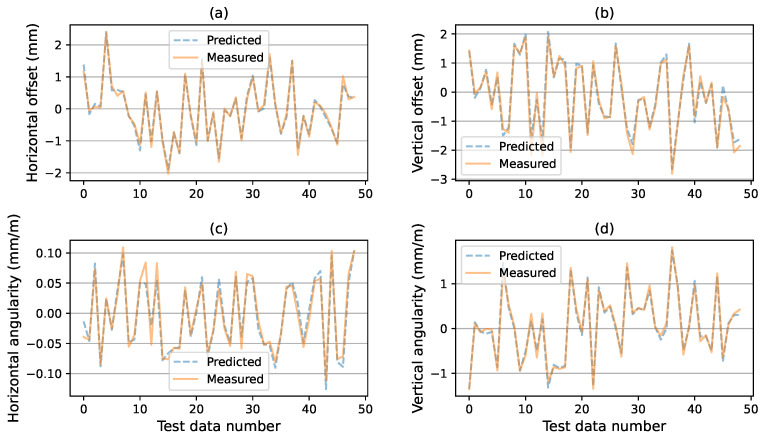
Comparision of the actual value with the predicted value of the proposed model based on GA-BP neural network. (**a**) comparison of predicted and measured horizontal offsets. (**b**) comparison of predicted and measured vertical offsets. (**c**) comparison of predicted and measured horizontal angularity. (**d**) comparison of predicted and measured vertical angularity.

(1) Performance results of the proposed prediction model. Using the training set and test set, the prediction model is trained and tested with the BP and GA-BP neural networks. We first trained the prediction model with GA-BP, and obtained the optimized weights and thresholds in the neural network as shown in Equations (24)–(27). According to these optimized parameters and Equation (1), we can obtain the explicit data relationship between the air spring pressure and alignment state for a specific ASVIS. And we can predict the alignment state by the collected air spring pressure values. Figure 6 shows the prediction results with the GA-BP neural network, in which 50 samples are randomly selected and displayed. We can see that all components of the alignment state can be accurately predicted. Thus, we can say that the alignment prediction model based on the GA-BP neural network has a good prediction ability.

(24) $$\left\{\omega_{n,k}^{i}\right\}=\left[\begin{array}{cccccccc} -1.01 & 0.41 & 0.49 & -0.45 & 0.00 & 0.01 & 0.05 & -0.79\\ 0.65 & 1.18 & 0.40 & 0.46 & -0.76 & 0.27 & -1.04 & 0.15\\ -0.24 & 0.80 & -0.79 & 0.42 & -0.34 & 0.01 & -0.24 & -0.21\\ -1.38 & -0.18 & 0.01 & -0.99 & -1.02 & 1.12 & -0.44 & -0.29\\ 0.86 & 1.08 & 0.28 & 0.39 & 0.50 & -0.39 & 0.64 & -0.73\\ -0.38 & 0.29 & -0.88 & 0.85 & 0.21 & 1.09 & 0.40 & 1.06\\ 0.22 & -0.95 & 0.12 & -0.21 & 0.34 & -1.18 & 0.03 & 0.04\\ 1.31 & -0.29 & -0.20 & -0.56 & 0.36 & -0.53 & 0.37 & -0.68 \end{array}\right]$$

(25) $$\left\{b_{k}^{i}\right\}=\left[\begin{array}{cccccccc} -0.36 & 0.92 & 1.42 & 0.66 & -0.96 & -0.71 & -0.51 & 0.72 \end{array}\right]$$

(26) $$\left\{\omega_{k,m}^{o}\right\}=\left[\begin{array}{cccccccc} -0.19 & 0.56 & 1.44 & -0.24 & -0.66 & 0.79 & 0.25 & -0.65\\ 0.13 & -1.10 & -0.89 & -0.21 & -0.23 & 1.47 & -0.65 & -0.38\\ -0.68 & 0.13 & -0.16 & -0.47 & -0.13 & 0.03 & 0.44 & 0.10\\ 0.47 & 1.39 & -0.42 & -0.79 & 0.87 & -0.46 & 0.29 & 0.20 \end{array}\right]$$

(27) $$\left\{b_{m}^{o}\right\}=\left[\begin{array}{cccc} -0.7121 & -2.6351 & 0.0761 & -1.2726 \end{array}\right]$$

To fully verify the effectiveness of the proposed model, we also tested the prediction model based on the BP neural network and GA-BP neural network with different performance metrics. As shown in Table 1, the prediction model performs well with both the BP and GA-BP neural networks, and the GA-BP neural network is superior to the BP neural network in these performance metrics. Compared with the original BP neural network, GA algorithm can help the prediction model reduce the MSE and MAE value by 74% and 38%, respectively, and increase the $R^{2}$ value by 12%. For single alignment state component, there is an interesting phenomenon. The horizontal angularity α performs best in MSE and MAE while it performs worst in $R^{2}$. In fact, this is because the value of α itself is small, which can be seen in Figure 5, so its MSE and MAE value are also small. However, consider that the determinant coefficient of α is the worst, the ability to predict and describe this component through the pressure value itself is the worst among all alignment state components. Besides, we test the prediction accuracy. And the accuracy of prediction model based on BP neural network and GA-BP neural network is 97.6% and 98.7%, in which GA-BP improves prediction accuracy by 1.1%. Thus, we can say that the introduction of GA algorithm in BP neural network can effectively improve the prediction accuracy and reduce the prediction error.

**Figure 7:**
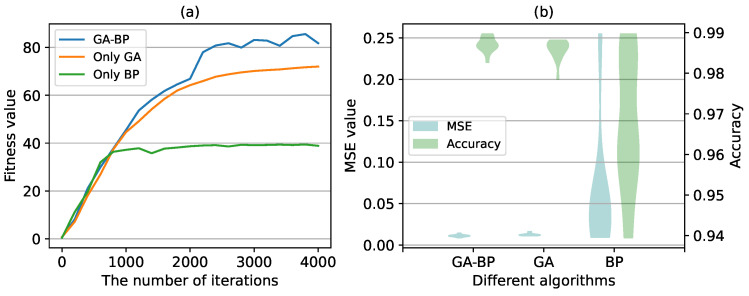
The prediction performance with different algorithms. (**a**) the fitness value in the training process with different algorithms. (**b**) the MSE value and accuracy of different algorithms calculated on the test set.

In addition, we compared the results of GA-BP neural network in terms of convergence and stability with different algorithms (i.e., GA and BP). Figure 7a shows the training process of the training set and Figure 7b shows the prediction performance of the test set, including MSE and accuracy. For the GA-BP neural network, GA is used to optimize network parameters in the first 2000 iterations, and BP algorithm is used to optimize network parameters in the last 2000 iterations. As shown in Figure 7a, compared with BP neural network, GA-BP neural network can obtain the global optimal value. Compared with only using GA to optimize network parameters, the convergence speed of GA-BP neural network is faster. As shown in Figure 7b, we can see that GA-BP and GA can stably obtain optimal values. However, the performance of the algorithm is unstable since the BP algorithm is easy to fall into local optimal.

**Figure 8:**
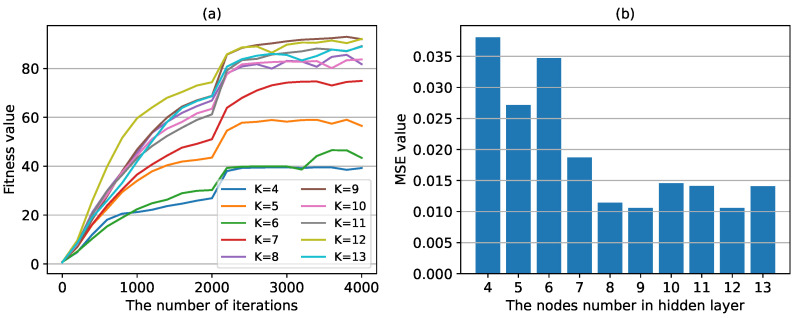
The fitness values and MSE values with different nodes number in the hidden layer. (**a**) the fitness values in the training process with different numbers of hidden layer nodes. (**b**) the MSE value calculated with the test set with different numbers of hidden layer nodes.

**Figure 9:**
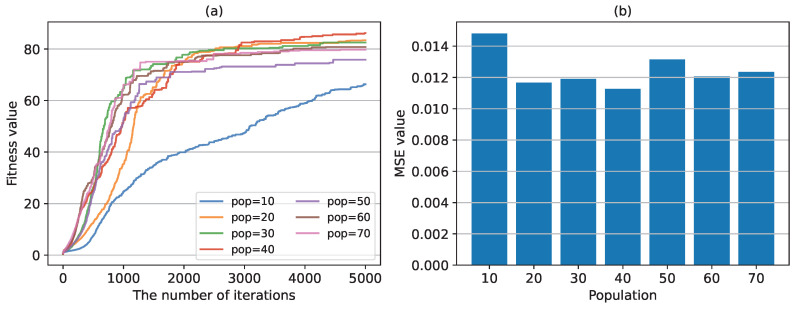
The fitness values and MSE values with different populations. (**a**) the fitness values in the training process with different populations. (**b**) the MSE value calculated for different populations in the test set.

(2) The impact of the number of nodes in hidden layer. The number of nodes in the hidden layer *K* is usually determined using an empirical equation, as shown in Equation (2). Here, we test the prediction performance of *K* in the range of 4∼13. Figure 8a shows the training process of prediction model based on GA-BP model, in which GA algorithm is used to optimize network parameters for the first 0–2000 iterations, and BP algorithm is used to optimize network parameters for the last 2000–4000 iterations. It can be seen that BP algorithm can quickly converge to the optimal value on the basis of the optimized parameters obtained by GA algorithm. And when K=8, the convergence rate of BP neural network has basically reached the maiximum. Figure 8b shows the MSE values calculated by the network model using optimized parameters on the test set. It can be seen that with the increase of the number of nodes in the hidden layer, the MSE value decreases, and when K=8, the prediction performance is basically optimal. This is consistent with the results shown in Figure 8a.

(3) The impact of different parameters in GA on the performance. We first test the effect of population size on the convergence speed and learning error of the GA algorithm. Figure 9a shows fitness value in the network parameters training process using the GA algorithm. The training process here does not include the iterative process of BP neural network. It can be seen that the population size affects the convergence speed of the algorithm. When the population size is 10, the convergence rate is the slowest. As the population size increases, the convergence speed increases. But when the population size reaches 40, the convergence rate is basically unchanged. Figure 9b shows the MSE values under different population sizes, which is calculated with the test set. Combined with the results in Figure 9a,b, it can be said that the population size has little effect on the learning error, mainly affecting the convergence speed of the GA algorithm.

**Figure 10:**
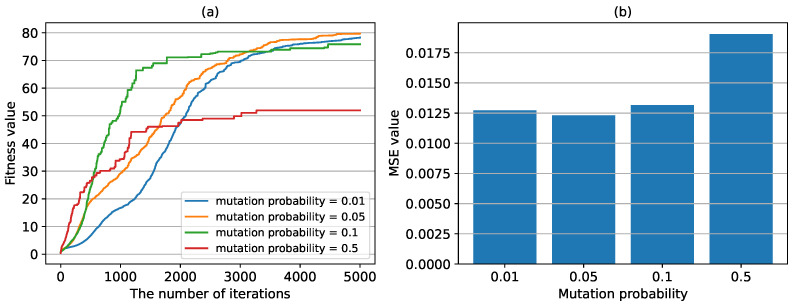
The fitness values and MSE values with different mutation probabilities. (**a**) the fitness values in the training process with different mutation probabilities. (**b**) the MSE value calculated in the test set with different mutation probabilities.

Then, we test the effect of mutation probability on the prediction performance. The mutation operation is mainly to avoid the algorithm falling into local optimum. We selected four probability values, namely 0.01, 0.05, 0.1 and 0.5, and the results are shown in Figure 10. It can be seen that when the mutation probability is too small, the convergence speed of the algorithm is slow. Increasing the mutation probability can improve the convergence speed of the algorithm. However, when the mutation probability is too high, it will affect the learning results, leading to the increase of MSE value.

**Figure 11:**
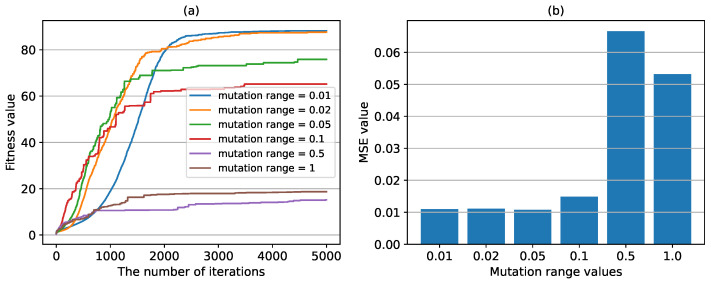
The fitness values and MSE values with different mutation ranges. (**a**) the fitness values in the training process with different mutation ranges. (**b**) the MSE value calculated in the test set with different mutation ranges.

Except for the mutation probability, the mutation range value can also affect the mutation operation in the GA algorithm, as shown in Equation (17). Here, we tested five different mutation range values, and the results are shown in Figure 11. It can be seen that the mutation range can affect the convergence speed and MSE value of the algorithm at the same time. The smaller the mutation probability, the slower the convergence speed, and the smaller the MSE value. However, when the mutation range value is reduced to 0.1, further reducing the value has little effect on the final learning result, i.e., MSE value.

## 5. Conclusions

In this paper, we investigate the alignment prediction problem in the air spring vibration isolation system, and present a data model to predict the alignment state using air spring pressures based on the BP neural network. And in order to obtain the global optimum, GA is applied to optimize the weights and thresholds of the BP neural network, and an improved GA-BP model is designed. To validate the effectiveness of the proposed alignment prediction model, we conducted the experiments in a real ASVIS, and four typical criteria, that is, MSE, MAE, R2, and accuracy, are introduced to evaluate the performance. The extensive experiment results show that the prediction model, based on the BP neural network and GA-BP neural network, can accurately predict the alignment state according to the air spring pressure. And compared with original BP neural network, GA-BP can achieve higher prediction accuracy and lower prediction error. In engineering, the prediction model proposed in this paper provides another approach to alignment prediction, which can be deployed independently or used as an assistant in case of sensor failure in alignment measurement, thus effectively improving the reliability of the system.

## Figures and Tables

**Table 1:** The results of proposed prediction model based on GA-BP and BP neural network.

Model		$x_{c}$	$z_{c}$	α	β	Total
GABP	MSE	0.0087	0.0287	0.0012	0.0072	**0.0114**
	MAE	0.0737	0.1253	0.0249	0.0662	**0.0725**
	R2	0.9902	0.9763	0.7477	0.9878	**0.9539**
BP	MSE	0.0845	0.0444	0.0037	0.0424	0.0438
	MAE	0.1384	0.1504	0.0493	0.1322	0.1176
	R2	0.905	0.9633	0.1963	0.9284	0.8545

## Data Availability

Data are unavailable due to privacy.

## Abbreviations

The following abbreviations are used in this manuscript:

ASVIS	air spring vibration isolation system
BP	back propagation
GA	genetic algorithm
NN	neural network
MAE	mean absolute error
MSE	mean-square error
ACU	air control unit
